# Decoy Effect on Consumers' Purchase Behaviors in Relation to Meat Products: Comparison of Pork and Chicken

**DOI:** 10.3389/fpsyg.2021.679256

**Published:** 2021-08-13

**Authors:** Lingling Xu, Meidan Yu, Xiujuan Chen

**Affiliations:** ^1^Institute for Food Safety Risk Management, School of Business, Jiangnan University, Wuxi, China; ^2^School of Business, Jiangnan University, Wuxi, China

**Keywords:** livestock and poultry meat products, food safety, animal welfare, decoy effect, bounded rationality

## Abstract

Few studies have analyzed the behaviors of consumers in relation to their purchase of meat products produced with animal welfare in consideration under different decoy scenarios; thus, it is difficult to accurately understand consumer behaviors and to reduce the bias in the conclusions of this study regarding consumption preferences in relation to meat products that had been produced with animal welfare in consideration. With the frequent occurrence of cases connected with the quality and safety of meat in China, the welfare conditions of livestock and poultry urgently need to be improved. We used 810 consumers in Wuxi City, Jiangsu Province, China as our study sample, chose pigs and chickens, i.e., the two common species of livestock and poultry, as study cases, and set four types of decoy scenarios based on breeding time, breeding model, diet cleanliness label, and price attributes, to explore the purchasing options of consumers for meat produced with high levels of animal welfare, under different decoy conditions. A decoy effect was observed in a bounded rational consumption situation in relation to the purchasing behaviors of both chicken and pork. In a situation of chicken purchase, the decoy effect of the breeding model was the strongest, followed by that of price, diet cleanliness label, and breeding time. In the case of pork purchase, the decoy effect of the diet cleanliness label was the strongest, followed by price, breeding model, and breeding time. In a comprehensive comparison between the two types of consumption experiments, price decoy constantly played a significant role, while the decoy effect of breeding time was the weakest. Accordingly, we proposed that in addition to strengthening the knowledge of people in the welfare of livestock and poultry, designing a breeding model decoy or price decoy in the process of chicken sales and designing a diet cleanliness label decoy or price decoy in the process of pork sales can guide the demand of consumers for meat produced with high levels of animal welfare. The welfare of livestock and poultry should thus be improved.

## Introduction

Scientific research has confirmed that among the infectious diseases that threaten human health, more than 200 infectious diseases are zoonoses and can be transmitted from animals to humans (World Health Organization, 2020), and among these zoonotic diseases, more than 130 have been found in China. In this study, live pigs were chosen as an example. The WHO and The Food and Agriculture Organization (FAO) have pointed out that there are six major infections in pigs, namely, swine fever, transmissible gastroenteritis, swine influenza, piglet paratyphoid, piglet colibacillosis, and swine pneumonia. Although swine fever can only infect pigs, anthrax can infect almost all mammals, including humans (Sheng, 2009). In recent years, outbreaks of new infectious diseases worldwide have become more frequent. These new infectious diseases have a common feature, in that they are related to animals to varying degrees and 70% of them are zoonotic (Iannetti et al., 2019). Although the causes of emerging zoonotic diseases are very complex, the disregard of people for animal welfare is a leading reason. A typical case is the influenza A (H1N1) global epidemic, which started in Mexico and the United States in 2009. In that year, the WHO confirmed a total of 29,080 cases of H1N1 in 79 countries and regions; 226 cases were confirmed in mainland China. The virus emerged from a pig farm in Mexico, where sewage flowed and where pigs were close-packed. Poor pig welfare contributed to the tragedy of the global pandemic (Wu, 2020). Thus, food quality and food safety are often linked to the food production methods (Harper and Henson, 1999), and poor animal welfare treatment, such as crowded environment and unclean diet, greatly increases the probability of animal diseases and the risk of their spreading from animals to humans through the food chain (European Food Safety Authority (EFSA), 2019; Iannetti et al., 2019). On the contrary, in the process of animal breeding and slaughtering, giving good animal welfare treatment can reduce the probability of animals suffering from diseases, enhance animal immunity, and improve the quality and safety of the meat (Gavinelli et al., 2007; Hartung et al., 2009; Velarde et al., 2015; Xu et al., 2019).

To suppress zoonotic diseases, reduce food safety issues, and improve the quality of meat products, animal welfare should be effectively protected in the process of livestock and poultry breeding, transportation, and slaughter (Zhao, 2010; Yang and Hong, 2019). However, improving the welfare of livestock and poultry would inevitably have an impact on the production costs and benefits for producers (Gocsik et al., 2016; Vissers et al., 2019). These measure whether to implement the production that takes welfare into account based on the economic feasibility of animal welfare plans and special market needs (Nocella, 2009; Mulder and Zomer, 2017). In China, consumers generally have low awareness of animal welfare, not realizing the internal connection between animal welfare protection and human health, and the market demand for meat produced meeting animal welfare needs is not sufficient to stimulate the welfare-related production behaviors among livestock and poultry producers (Gu, 2017; Ma, 2019). Thus, most livestock and poultry farmers generally ignore animal welfare issues in their pursuit of production efficiency and profit maximization, resulting in frequent outbreaks of quality and safety issues in relation to various meat products (Hartung et al., 2009; Deng and Xiao, 2017). To improve the welfare of livestock and poultry animals in China, it is thus necessary to scientifically formulate the marketing strategies that would expand the market demand for livestock and poultry products with animal welfare attributes taken into consideration based on the consumer preferences to stimulate the welfare production behaviors among livestock and poultry producers.

Using the traditional rational choice theory, scholars have studied the preferences of consumers and their willingness to pay for animal welfare-related attributes in the production of livestock and poultry products, i.e., whether consumers would choose animal welfare-friendly livestock and poultry products with maximum utility under their budget constraints. However, the context effect based on the modern decision-making theory overturns the traditional rational choice theory, indicating that the context has a systemic influence on the preferences and choices of consumers, thus leading to the bounded rationality of the decision-making behaviors of consumers (Bettman et al., 1998; Novemsky et al., 2007; Sun et al., 2017). The decoy effect is a context effect, i.e., after adding new options to a commodity selection set, the probability of an alternative option in the commodity selection set being selected by consumers will increase (Huber et al., 1982; Brenner et al., 1999; Park and Kim, 2005). Heath and Subimal (1995) found that the decoy effect is pervasive in consumer preference. Analyzing the decoy effect can help us understand consumer behaviors more accurately and reduce the deviations in consumer preference in study conclusions (Trueblood et al., 2014; Noguchi and Stewart, 2018). However, few studies have analyzed the decoy effect in the consumption behavior of consumers in relation to livestock and poultry products that had been produced with animal welfare attributes in consideration from the perspective of decision-making under bounded rationality. To better assist companies market livestock and poultry products with animal welfare attributes that were taken into account, this study used yellow-feathered broilers and pork hindquarters as cases and introduced decoy scenarios based on the animal welfare attributes to analyze whether there exists a decoy effect in the consumption behaviors of consumers in relation to livestock and poultry products with animal welfare attributes taken into account and to compare the decoy effect strength of animal welfare attributes of different products.

## Literature Review and Hypotheses

**Figure 1:**
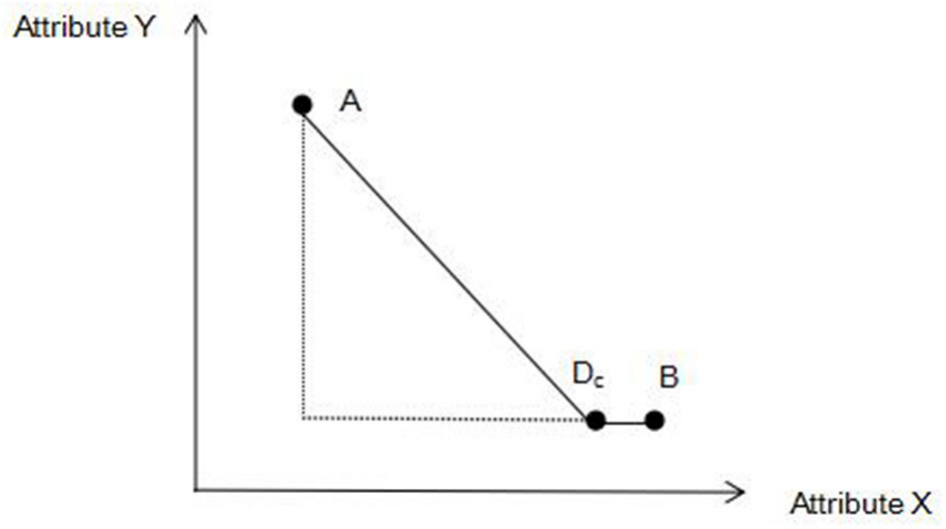
Schematic diagram of the mechanism of decoy effect.

The emergence of decoy products highlights the pros and cons of options in a selection set (Brenner et al., 1999), which can help decision-makers reduce cognitive load and simplify decision-making (Nowlis et al., 2010). From the perspective of value transfer, decoy products change the attribute ranking of selected products, making the target products has a comparative advantage (Pettibone and Wedell, 2000). As shown in Figure 1, in the core set containing products *A* and *B*, a decoy product *D*_*C*_ with a subjective value similar to product *B* is set. Compared with product B, if the decoy product *D*_*C*_ has utility benefit on the attribute *Y* but has utility loss on the attribute *X*, consumers may choose product *B* based on simplified decision-making and loss aversion. Thus, when the optimal decoy product is designed, the high-quality performance of the main product can be significantly highlighted (Herweg et al., 2017), and scholars have carried out studies on how the decoy effect affects the purchasing decision of consumers and its strength.

Many factors affect the decoy effect, but Müller et al. (2014) believed that the most basic condition for the occurrence of a positive decoy effect is the ability of a decoy product to trigger consumer trade-offs and comparisons with the target products. Therefore, consumers who rely on intuitive reasoning (Mao and Oppewal, 2012) and who use the intuitive information processing mode (Mourali et al., 2007) are more likely to generate a decoy effect. Hamilton et al. (2007) found that when the number of product attributes is large, the consideration of consumers for all attributes would be difficult, and a few attributes with higher utility would have a stronger decoy effect. Scarpi (2008) focused on analyzing the strength of the attraction effect under the situations of background information, real decoy^1^, and phantom decoy^2^ and also found that when the decoy situation and the background situation contrasted in guiding consumer behaviors, the decoy effect was more effective, and the attraction effect of the phantom decoy was stronger than that of the real decoy. Frederick et al. (2014) used lottery games and television purchases as examples and found that when the product attributes were represented as graphics, shaded areas, and other perceptions that could be directly experienced, the decoy effect might be reduced, eliminated, or even reversed, while only when the product attributes were represented by numbers, grades, and other similar concepts, the decoy effect was relatively obvious. Zhang and Liu (2017) used fitness packages as an example and found that increasing a fixed fee decoy for deterministic payment and an extra fee decoy for uncertain payment in a package service increases the probability of the target alternative being selected, but the fixed fee decoy for deterministic payment had a stronger attracting effect. Schumpe et al. (2020) found that setting text prompts as a decoy before shopping advertisements can divert the resistance of consumers to shopping advertisements, which is manifested as a greater willingness to purchase. Wu et al. (2020) used traceable pork hindquarters as a study case and found that when consumers made consumption choices in a non-induced situation and then made choices in a situation with inductive information, the decoy effect was relatively stronger, while the direct creation of the decoy situation had a relatively small impact on the purchase selection behaviors of consumers.

Nonetheless, some studies have shown that decoys may fail and even have a resistance effect in the process of the purchases of consumers. For example, when consumers are more knowledgeable about a product, or purchase products based on the experience rather than product descriptions, they are less affected by the decoy situation (Dhar and Glazer, 1996; Putrevu and Lord, 2001; Mourali et al., 2007; Hadar et al., 2018). Costanigro and Lusk (2014) used food labels that indicated ethylene ripening in food as decoys to explore the acceptance of consumers of food labels that indicate the genetic modification. The results showed that prior exposure to such labels did not lead to significant differences in consumer aversion to genetically modified foods. Attwood et al. (2020) used high-priced vegetarian food as a disadvantage decoy to induce consumers to choose a sustainable vegetarian diet. Their findings showed that the addition of the high-priced vegetarian decoy did not significantly change the number of consumers who chose the target vegetarian food and competitive meat. The reason for this may be that the price decoy set in the experiment failed to inspire and induce the utility weighing mechanism of consumers. Similarly, Ohlhausen and Langen (2020) compared the attractiveness of descriptive dish labels with that of decoy options and found that labels can positively affect the sustainable consumption of consumers, while decoy dishes reduce the probability of choosing target dishes. A possible reason for this may be that the decoy attributes failed to make consumers perceive the utility differences between the target and competing dishes. In addition, Rogers et al. (2020) have also attempted to use decoy programs to encourage smokers to participate in a specified number of smoking cessation meetings, but the time decoy failed to guide smokers to increase their choice of target programs. It could be that the decoy strength was too low or that quitters saw “attendance time” not as an opportunity cost but as a benefit. Evidently, successful decoys should be able to influence the relative value judgment of consumers on the attributes of selected products and induce them to choose appropriate target products based on the value judgment mechanism.

In summary, for consumer purchase behaviors, decoy products may trigger a positive decoy effect or cause decoy failure. So far, there have been few studies on consumer behaviors in relation to livestock and poultry products with animal welfare taken into consideration under different decoy scenarios; thus, it is difficult to provide a scientific basis for the marketing of livestock and poultry produced with animal welfare in consideration. Hence, this study took two common meat products, namely, yellow-feathered broilers and pork hindquarters, as examples and designed decoy products according to different attributes (including three attributes related to animal welfare, namely, the breeding time, breeding model, and diet cleanliness label, as well as price attributes). The purpose was to explore whether these four types of attributes have a decoy effect on the purchase behavior of consumers concerning livestock and poultry meat products and to compare the value difference of the decoy effect of the same attribute in different purchase situations of livestock and poultry products. Based on the above considerations, the assumptions were made as follows:

H1a: The attribute of breeding time does not have a positive decoy effect on the chicken purchase of consumers.
H1b: The attribute of breeding time does not have a positive decoy effect on the pork purchase of consumers.
H2a: The attribute of breeding model does not have a positive decoy effect on the chicken purchase of consumers.
H2b: The attribute of breeding model does not have a positive decoy effect on the pork purchase of consumers.
H3a: The attribute of diet cleanliness label does not have a positive decoy effect on the chicken purchase of consumers.
H3b: The attribute of diet cleanliness label does not have a positive decoy effect on the pork purchase of consumers.
H4a: The price attribute does not have a positive decoy effect on the chicken purchase of consumers.
H4b: The price attribute does not have a positive decoy effect on the pork purchase of consumers.

## Experimental Design, Implementation, and Sample Characteristics

We applied the following experimental design and launched a specific implementation plan to achieve our experimental goal.

### Experimental Design

#### Setting of Experimental Subjects

Among livestock and poultry products, Chinese consumers generally prefer pork and chicken. According to the US Department of Agriculture (USDA), in 2019, the pork and chicken production in China reached 42.55 million tons and 13.80 million tons, respectively, accounting for 43 and 14% of the total global pork and chicken production, respectively. Therefore, we chose chicken and pork as our experimental products. In addition, to avoid the interference of different meat sources, types, and parts of livestock and poultry on the experimental results, we chose domestic yellow-feathered broilers and domestic pork hindquarters as our specific varieties for our experiment and informed the interviewees before the experiment.

#### Attribute Setting of Livestock and Poultry Meat Products

Considering that the current welfare problems concerning livestock and poultry in China are mainly concentrated on excessively fast breeding times, high breeding densities, and unclean feeding (Yang et al., 2016; Deng and Xiao, 2017; Zhang et al., 2018), we set breeding time, breeding model, diet cleanliness label, and price as four attributes for yellow-feathered broilers and pork hindquarters, as shown in Table 1. According to our preliminary investigation, the growth time of yellow-feathered broilers ranges from 2 to 4 months, and the growth time of live pigs ranges from 6 to 10 months. A very short growth time can easily burden the heart and lungs of livestock and poultry, which is not conducive to animal welfare (Carlsson et al., 2007; Mulder and Zomer, 2017). Therefore, the breeding time levels of yellow-feathered broilers were set as *fast* (2 months) and *slow* (4 months), and those of pork hindquarters were set as *fast* (6 months) and *slow* (10 months). A relaxed environment where animals can eat, drink, and move freely also reduces the risk of depression and lameness (Carlsson et al., 2007; de Graaf et al., 2016; Wolf and Tonsor, 2017). Thus, the breeding model levels of yellow-feathered broilers were set as *cage breedin*g^3^ and *free-range breeding*^4^, and those of pork hindquarters were set as *limited-fence breedin*g^5^ and *free-range breeding*^6^. To ensure the diet-related health of livestock and poultry, drinking water, and feed should comply with the China GB 5749 and NY/T 5027 standards. Thus, the levels of diet cleanliness label for yellow-feathered broilers and pork hindquarters were set as *with diet cleanliness label* and *without diet cleanliness label*. Referring to the average retail prices in large supermarkets and e-commerce platforms in 2019, the price of ordinary domestic yellow-feathered broilers was about 15 yuan per catty, while their price can reach 30 yuan per catty if they meet a slaughter time of more than 4 months and are raised freely. Thus, the price attribute levels for yellow-feathered broilers in this study were set at 15 yuan/catty, 22.5 yuan/catty, and 30 yuan/catty. In the same period, the retail price of ordinary domestic pork hindquarters was about 22 yuan per catty, while their price can reach 40 yuan per catty if they satisfy the welfare breeding conditions such as slaughter for more than 10 months and moderate free-range breeding. Thus, the price attribute levels for pork hindquarters in this study were set at 22 yuan/catty, 31 yuan/catty, and 40 yuan/catty.

**Table 1:** The setting of the attributes and their corresponding levels.

**Attributes**	**The levels for chicken**	**The levels for pork**
	**(yellow-feathered broiler)**	**(pork hindquarter)**
Breeding time	*Fast* (2 months)	*Fast* (6 months)
	*Slow* (4 months)	*Slow* (10 months)
Breeding model	*Cage breeding*	*Limited-fence breeding*
	*Free-range breeding*	*Free-range breeding*
Diet cleanliness label	*Without*	*Without*
	*With*	*With*
Price (yuan/catty)	*15*	*22*
	*22.5*	*31*
	*30*	*40*

#### Experimental Design

As shown in Table 2, in the chicken consumption experiment, to explore the choice of consumers of high welfare chicken under decoy conditions or not, we referred to the study by Wu et al. (2020) and set three welfare levels for yellow-feathered broilers as low, medium, and high, respectively, represented by *a, b*, and *c*, which constitute the core set *U*{*a, b, c*}, in which option *c* is the target product^7^, and options *a* and *b* are competitive products^8^. Four types of decoy options were separately set up based on the attributes of breeding time, breeding model, diet cleanliness label, and price, which are represented by *d, e, f*, and *g* and form the extension set *U*_1_{*a, b, c, d*}, *U*_2_{*a, b, c, e*}, *U*_3_{*a, b, c, f*}, and *U*_4_{*a, b, c, g*}. Among these, chicken *c* is superior to chickens *d, e, f*, and *g* in terms of breeding time, breeding model, diet cleanliness label, and price, respectively. In the same way, in the pork hindquarter consumption experiment, we set three welfare levels for pork hindquarters as low, medium, and high, represented by *h, i*, and *j*, which constitute the core set *V*{*h, i, j*}, in which option *j* is the target product, and options *h* and *i* are competitive products. We set up four types of decoy options based on the attributes of breeding time, breeding model, diet cleanliness label, and price, which are separately represented by *k, l, m*, and *n* and form the extension sets *V*_1_{*h, i, j, k*}, *V*_2_{*h, i, j, l*}, *V*_3_{*h, i, j, m*}, and *V*_4_{*h, i, j, n*}. Among these, pork *j* is superior to pork *k, l, m*, and *n* in terms of breeding time, breeding model, diet cleanliness label, and price, respectively (Table 2).

**Table 2:** Consumption options of livestock and poultry products.

**Types**	**Options**
Yellow-feathered broiler	Fast (2 months), cage breeding, without diet cleanliness label, 15 yuan/catty (a)
	Fast (2 months), cage breeding, with diet cleanliness label, 22.5 yuan/catty (b)
	Slow (4 months), free-range breeding, with diet cleanliness label, 30 yuan/catty (c)
	Fast (2 months), free-range breeding, with diet cleanliness label, 30 yuan/catty (d)
	Slow (4 months), cage breeding, with diet cleanliness label, 30 yuan/catty (e)
	Slow (4 months), free-range breeding, without diet cleanliness label, 30 yuan/catty (f)
	Slow (4 months), free-range breeding, with diet cleanliness label, 33 yuan/catty (g)°
Pork hindquarter	Fast (6 months), limited-fence breeding, without diet cleanliness label, 22 yuan/catty (h)
	Fast (6 months), limited-fence breeding, with diet cleanliness label, 31 yuan/catty (i)
	Slow (10 months), free-range breeding, with diet cleanliness label, 40 yuan/catty (j)
	Fast (6 months), free-range breeding, with diet cleanliness label, 40 yuan/catty (k)
	Slow (10 months), limited-fence breeding, with diet cleanliness label, 40 yuan/catty (l)
	Slow (10 months), free-range breeding, without diet cleanliness label, 40 yuan/catty (m)
	Slow (10 months), free-range breeding, with diet cleanliness label, 44 yuan/catty (n)*^*p*^*

*To compare the decoy effect of the price attributes of the two types of livestock and poultry products, the price decoy was set to be 10% higher than the price of the target product; thus, o: 30 × (1 + 10%) = 33; p: 40 × (1 + 10%) = 44*.

In the yellow-feathered broiler consumption experiment, observing the purchase share of consumers for *c* in the core set *U* and the four extension sets, compared with the purchase share of *c* in the core set, if the relative purchase share of target product *c* to competing products *a* and *b* does not increase in the extension set, which includes a certain decoy based on the corresponding attribute, then the decoy product set based on this attribute does not have a positive decoy effect on consumer purchase behavior; the hypotheses H1a to H4a are then tested accordingly. In the same way, in the pork hindquarter consumption experiment, observing the purchase shares of consumers for *j* in the core set *V* and the four expansion sets, compared with the purchase share of *j* in the core set, if the relative purchase share of target product *j* to competing products *h* and *i* does not increase in the extension set, which includes a certain decoy based on the corresponding attribute, then the decoy products set based on this attribute do not have a positive decoy effect on consumer purchase behavior, and the hypotheses H1b to H4b are tested accordingly. On this basis, the decoy effect strength of the same attribute in the purchase process of different livestock and poultry meat products is compared.

### Organizational Implementation and Sample Characteristics

#### Experimental Organization and Questionnaire Survey

The experimental and questionnaire investigation was conducted in Wuxi, Jiangsu Province, China. According to the data from the National Bureau of Statistics in 2019, Wuxi, a city with an area of 4,627.46 square kilometers and a permanent resident population of 6.59 million, is one of the most densely populated cities in the Yangtze River Delta region (>1,400 permanent residents/square kilometers). The per capita gross domestic product of Wuxi ranks second among Chinese cities, its overall level of economic and social development is in the lead, and both the awareness of food safety consumption of the residents and the demand for food safety information are relatively strong. In addition, Wuxi has one of the largest meat distribution centers (Tianpeng Food City) in East China; hence, it is also a key city for food marketing and food safety supervision. Wuxi thus provides a good foundation to carry out studies such as the present study. To ensure the representativeness of the experimental sample, trained postgraduate students from a local university acted as investigators and randomly recruited participants in large supermarkets and butcher markets from all five administrative areas of urban Wuxi. During recruitment, every third consumer participated as experiment participants (Wu et al., 2012). For the sake of simplicity, an equal number of adults (162 in total) aged between 18 and 65 years were recruited from each administrative area: 81 participants for the chicken consumption experiment and 81 participants for the pork consumption experiment. The experiments were completed in five batches from June 1 to June 20, 2020. In addition to the consumption experiment part of the questionnaire, the participants also need to answer questions such as individual characteristics in the questionnaire. Finally, a total of 810 questionnaires were obtained: 405 for the yellow-feathered broiler experiment and the pork hindquarter experiment, respectively. To improve their enthusiasm to participate, each participant who completed the experiment was given a small gift as compensation for their time.

#### Sample Characteristics

The sample characteristics are shown in Table 3. The proportion of women in the sample was 55.3%, which is consistent with the fact that women are more likely to buy food in Chinese families. Of the participants, 77.8% aged below 40 years, 66.2% had a college- or university-level education, and 62% were urban residents. The monthly personal income of them was mainly concentrated in the two levels of 3,000–6,000 yuans and 6,000–8,000 yuans, accounting for 27.5 and 37.2% of the total consumers, respectively. Among the 810 respondents, only 2.6 and 0.3% of them had a “high” or “very high” knowledge of animal welfare, and the knowledge of most respondents of animal welfare was “low” or “very low,” indicating that the understanding of animal welfare among Chinese consumers is inadequate.

**Table 3:** Demographic characteristics of respondents.

**Statistical indicator**	**Category**	**Yellow-feathered broiler**	**Pork hindquarter**	**Total**	
		**Frequency**	**Frequency**	**Frequency**	**Effective percentage (%)**
Gender	Male	187	175	362	44.7
	Female	218	230	448	55.3
Age (year)	18–30	191	213	404	49.9
	31–40	116	110	226	27.9
	41–50	79	69	148	18.3
	51–65	19	13	32	3.9
Level of education	Primary school and below	10	8	18	2.2
	Junior high school	40	37	77	9.5
	Senior high school (including technical secondary school)	66	67	133	16.4
	College	89	105	194	24.0
	University	173	169	342	42.2
	Postgraduate and above	27	19	46	5.7
Address	Urban	259	243	502	62.0
	Rural	146	162	308	38.0
Personal monthly income	≤ 3,000 yuan	54	45	99	12.2
	3,001–6,000 yuan	114	109	223	27.5
	6,001–8,000 yuan	154	147	301	37.2
	8,001–12,000 yuan	58	79	137	16.9
	>12,000 yuan	25	25	50	6.2
Knowledge of animal welfare	Very low	98	108	206	25.4
	Low	193	229	422	52.1
	Medium	101	58	159	19.6
	High	12	9	21	2.6
	Very high	1	1	2	0.3

## Analysis Framework, Results, and Discussion

### Analysis Framework

For the yellow-feathered broiler consumption experiment, based on the calculation method of Mourali et al. (2007), we set *P*(*c*; *U*) as the absolute purchase share of target product *c* in the core set, while *P*(*a*; *U*_1_), *P*(*b*; *U*_1_), *P*(*c*; *U*_1_), and *P*(*d*; *U*_1_) refers to the absolute purchase share of competitive products *a* and *b*, target product *c*, and decoy product *d* in the extension set *U*_1_{*a, b, c, d*}, respectively. *P*_*d*_(*c*; *a, b*) refers to the relative purchase share of competing products *a* and *b* in the expansion set *U*_1_{*a, b, c, d*} after adding the decoy product *d*.

(1) $$\begin{array}{l} P_{d}\left(c;a,b\right)=\frac{P\left(c;U_{1}\right)}{P\left(a;U_{1}\right)+P\left(b;U_{1}\right)+P\left(c;U_{1}\right)}=\frac{P\left(c;U_{1}\right)}{1-P\left(d;U_{1}\right)} \end{array}$$

The value of the decoy effect is represented by Δ*P* as follows:

(2) $$\begin{array}{l} \Delta P=P_{d}\left(c;a,b\right)-P\left(c;U\right) \end{array}$$

When Δ *P* > 0, a positive decoy effect occurs; when Δ*P* < 0, a negative decoy effect occurs. The formula for the strength coefficient of the decoy effect is as follows:

(3) $$\begin{array}{l} K=\frac{P_{d}\left(c;a,b\right)}{P\left(c;U\right)} \end{array}$$

The greater the *K* value, the greater the strength of the decoy effect.

### Results of the Chicken Experiment With Different Welfare Levels

As shown in Tables 4, 5, in the yellow-feathered broiler core set *U*{*a, b, c*}, the absolute shares of consumers that chose yellow-feathered broilers *a, b*, and *c* were 10.1, 37.8, and 52.1%, respectively, among which *c* refers to the target product; thus, *P*(*c*; *U*) = 52.1%. In the expansion set *U*_1_{*a, b, c, d*}, after adding the breeding time decoy *d*, the absolute share of consumers that chose yellow-feathered broiler *c* was 59.8%. According to formulas (1)–(3), the relative purchase share *P*_*d*_(*c*; *a, b*) = 62.2%, *P*(*c*; *U*) < *P*_*d*_(*c*; *a, b*), the Δ*P* value of the decoy effect of the breeding time attribute was 10.1%, and the strength *K* was 1.19. Therefore, hypothesis H1a is rejected, and the attribute of breeding time has a positive decoy effect on the chicken purchase of consumers. In the expansion set *U*_2_{*a, b, c, e*}, after adding the breeding method decoy *e*, the absolute share of consumers that chose yellow-feathered broiler *c* was 62.5%, *P*_*e*_(*c*; *a, b*) = 65.6%, *P*(*c*; *U*) < *P*_*d*_(*c*; *a, b*), the Δ*P* value of the decoy effect of the breeding time attribute was 13.5%, and the strength *K* was 1.26. Therefore, hypothesis H2a is rejected, and the attribute of the breeding model has a positive decoy effect on the chicken purchase of consumers.

**Table 4:** The absolute purchase share in the yellow-feathered broilers experiment.

**Product options**	**Core set U**	**Extension set**			
		**U_**1**_**	**U_**2**_**	**U_**3**_**	**U_**4**_**
a	41 (10.1%)	24 (5.9%)	22 (5.4%)	21 (5.2%)	25 (6.2%)
b	153 (37.8%)	123 (30.4%)	111 (27.4%)	124 (30.6%)	109 (26.9%)
c	211 (52.1%)	242 (59.8%)	253 (62.5%)	241 (59.5%)	251 (62.0%)
d	–	16 (3.9%)	–	–	–
e	–	–	19 (4.7%)	–	–
f	–	–	–	19 (4.7%)	–
g	–	–	–	–	20 (4.9%)

**Table 5:** Δ*P* and *K* of decoy effect in the yellow-feathered broilers experiment.

**Variables**	**Extension set**			
	**U1**	**U2**	**U3**	**U4**
Δ*P* (χ^2^(3))	10.1%^*^ (25.83)	13.5%^*^ (35.21)	10.3%^*^ (30.48)	13.1%^*^ (34.73)
*K*	1.19	1.26	1.20	1.25

* *Significant at 1% significance level*.

Similarly, in the extension set *U*_3_{*a, b, c, f*} with the diet cleanliness label decoy *f* and the extension set *U*_4_{*a, b, c, g*} added with the price decoy *g*, the absolute shares of consumers that chose yellow-feathered broiler *c* were 59.5 and 62.0%, *P*_*f*_(*c*; *a, b*) = 62.4%, *P*_*g*_(*c*; *a, b*) = 65.2%; *P*(*c*; *U*) < *P*_*f*_(*c*; *a, b*), *P*(*c*; *U*) < *P*_*g*_(*c*; *a, b*), the Δ*P* values of the decoy effect of the breeding time attribute were 10.3 and 13.1%, and the strength *K* were 1.20 and 1.25, respectively. Therefore, hypotheses H3a and H4a are rejected: diet cleanliness label and price have a positive decoy effect on the chicken purchase behavior of consumers.

It can thus be noted that in the yellow-feathered broiler consumption experiment, all the four attributes we investigated could trigger a decoy effect, with the order of strength as follows: breeding model > price > diet cleanliness label > breeding time. A possible reason is that the traditional way of raising chickens in China is pasture-rearing, and Chinese consumers are more satisfied with the nutritional value and taste of free-range chickens (Zhu et al., 2019); thus, the decoy of the breeding model had the strongest effect. The decoy effect of the price attribute was also relatively strong. Whether the decoy strength of the effective attribute or price of the products is prominent depends on the value comparison of each attribute and target attribute in the consumer choice set (Herweg et al., 2017), which shows that consumers are of great concern to the price of high-welfare meat products. For Chinese consumers, compared with the free-range raising model, the cleanliness of the diet has a lower impact on the taste of broilers and the level of animal welfare. Therefore, the decoy effect of the cleanliness label attribute was not high, ranking third. The decoy effect of the breeding time attribute was the weakest, possibly because there have been few reports of chicken safety incidents related to broiler breeding time; hence, the attribute may have not yet been attracted the attention of consumers.

### Results of Pork Hindquarter Experiment With Different Welfare Levels

As shown in Tables 6, 7, in the core set *V*{*h, i, j*}, the absolute shares of consumers that chose pork hindquarters *h, i*, and *j* were 10.6, 31.9, and 57.5%, respectively, where *j* is the target product, so *P*(*j*; *V*) was 57.5%. In the expansion set *V*_1_{*h, i, j, k*}, after adding the breeding time decoy *k*, the absolute share of consumers that chose pork hindquarter *j* was 64.0%. Thus, the relative purchase share *P*_*k*_(*j*; *h, i*) was 67.5%, *P*(*j*; *V*) < *P*_*k*_(*j*; *h, i*), the Δ*P* value of the decoy effect of the breeding time attribute was 10.0%, and the strength *K* was 1.17. Therefore, hypothesis H1b is rejected, and the attribute of breeding time has a positive decoy effect on the pork purchase of consumers. In the expansion set *V*_2_{*h, i, j, l*}, after adding the breeding time decoy *l*, the absolute share of consumers that chose pork hindquarter *j* was 64.9%, *P*_*l*_(*j*; *h, i*) = 68.0%, *P*(*j*; *V*) < *P*_*l*_(*j*; *h, i*), the Δ*P* value of the decoy effect was 10.5%, and the strength *K* was 1.18. Therefore, hypothesis H2b is rejected, and the attribute of the breeding model has a positive decoy effect on the pork purchase of consumers.

**Table 6:** The absolute purchase share in the pork hindquarter experiment.

**Product options**	**Core set V**	**Extension set**			
		**V_**1**_**	**V_**2**_**	**V_**3**_**	**V_**4**_**
a	43 (10.6%)	39 (9.6%)	39 (9.6%)	33 (8.1%)	32 (7.9%)
b	129 (31.9%)	86 (21.2%)	85 (21.0%)	79 (19.5%)	81 (20.0%)
c	233 (57.5%)	259 (64.0%)	263 (64.9%)	277 (68.4%)	273 (67.4%)
d	–	21 (5.2%)	–	–	–
e	–	–	18 (4.5%)	–	–
f	–	–	–	16 (4.0%)	–
g	–	–	–	–	19 (4.7%)

**Table 7:** Δ*P* and *K* of decoy effect in the pork hindquarter experiment.

**Variables**	**Extension set**			
	**V1**	**V2**	**V3**	**V4**
Δ*P* (χ^2^(3))	10.0%^*^ (31.17)	10.5%^*^ (29.06)	13.7%^*^ (33.13)	13.2%^*^ (34.75)
*K*	1.17	1.18	1.24	1.23

* *Significant at 1% significance level*.

Similarly, in the extension set *V*_3_{*h, i, j, m*} with the diet cleanliness label decoy *m* and the extension set *V*_3_{*h, i, j, n*} with the price decoy *n*, the absolute shares of consumers that chose pork hind leg *j* were 68.4 and 67.4%, respectively. *P*_*m*_(*j*; *h, i*) = 71.3%, *P*_*n*_(*j*; *h, i*) = 70.7%, *P*(*j*; *V*) < *P*_*m*_(*j*; *h, i*), *P*(*j*; *V*) < *P*_*n*_(*j*; *h, i*), the Δ*P* values of the decoy effect were 13.8 and 13.2%, and the strengths were 1.24 and 1.23, respectively. Therefore, hypotheses H3b and H4b are rejected: the diet cleanliness label and price have a positive decoy effect on the pork purchase of consumers.

It can be noted that in the pork hindquarter consumption experiment, all four attributes had a decoy effect, and the order of strength was as follows: diet cleanliness label > price > breeding model > breeding time. In the traditional livestock and poultry breeding models of China, pigs are generally raised in captivity. Thus, the strength of the decoy effect of the breeding model of pigs did not rank first but ranked third. The outbreak of African swine fever in recent years has caused Chinese consumers to panic about the safety of meat. Swill feeding can spread mycotoxins in pigs. It is one of the inducements for African swine fever, and Chinese consumers find it disgusting. Hence, the attribute of diet cleanliness labels could trigger a stronger decoy effect. This point is similar to the conclusion that Liu and Chen (2019) made in their study on the decoy effect in traceable pork hindquarters, i.e., the attribute that reflects the quality and safety information of pork has a stronger decoy effect. The decoy strength of price ranked second in the results of the yellow-feathered broiler consumption experiment, indicating that the impact of price on consumer buying behavior is always relatively important. This shows that in the promotion of animal welfare meat products, the price factor constantly plays an important role. Compared with the decoy effects of other attributes, the decoy effect of breeding time ranked last. A possible reason is that compared with the clean diet and breeding model, the attribute of breeding time has a relatively small impact on the welfare of pigs and pork quality or safety, thus reducing the intensity of the decoy.

### Comparison and Discussion of the Results of the Two Types of Livestock and Poultry Product Consumption Experiments

**Figure 2:**
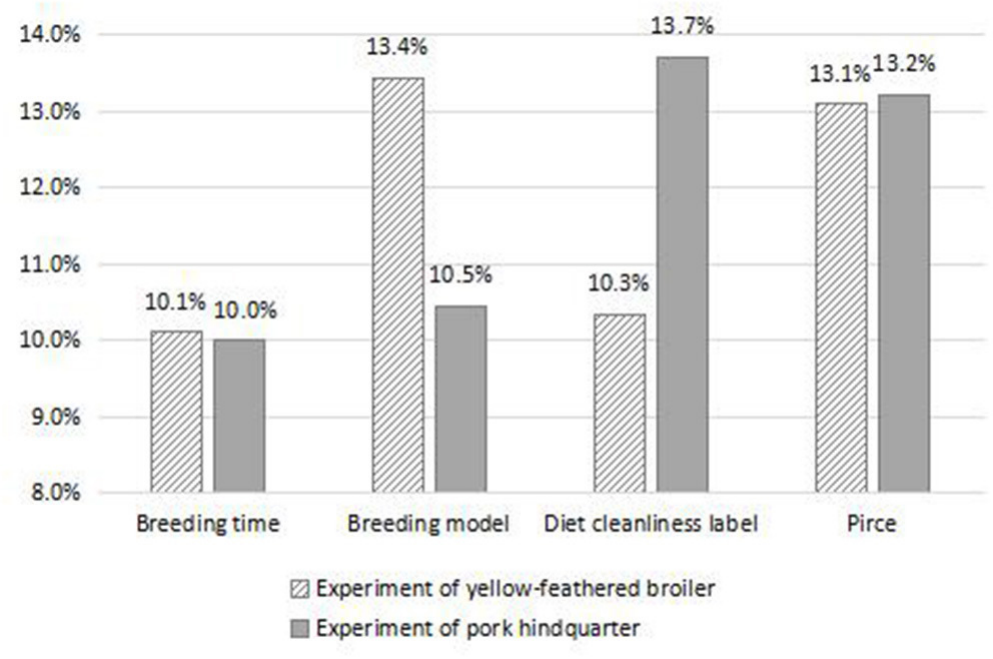
Δ*P* of decoy under different attributes in the two experiments.

As illustrated in Figure 2, it can be noted that there are differences in the value of the decoy effect of different attributes in the two experiments. The Δ*P* value of the price decoy ranked second, i.e., 13.1 and 13.2% for the chicken and pork experiments, respectively; that is, in the process of guiding consumers to purchase livestock and poultry meat products with the high-level animal welfare, it is effective to set decoy products based on the price attributes. The effect of the breeding time decoy was relatively lower and ranked at the bottom, i.e., 10.1 and 10.0% for the chicken and pork experiments, respectively. The premise of the existence of the decoy effect is that the properties can trigger the trade-off of target products of consumers. Thus, consumers pay less attention to the growth time of livestock and poultry in their purchase decisions of livestock and poultry produced with animal welfare in consideration. The difference is that first, in the yellow-feathered broiler consumption experiment, the decoy effect of the breeding model was the largest, reaching 13.4%, while in the pork hindquarter consumption experiment, the decoy effect of the breeding model was not high, only 10.5%. It may be that in Chinese traditional livestock and poultry farming models, chickens are mostly raised freely, and pigs are mostly raised in captivity; hence, the value of the decoy effect of the broiler breeding model is significantly higher than that of live pigs. Second, in the yellow-feathered broiler consumption experiment, the value of the decoy effect of the diet cleanliness label was only 10.3%, while that in the pork hindquarter consumption experiment reached up to 13.7%. This may be that due to the frequent exposure of negative news regarding “swill pigs” and “clenbuterol pigs” and the negative impact of African swine fever, consumers are more concerned about the cleanliness of the diet of pigs.

## Conclusion and Implications

This study took yellow-feathered broilers and pig hindquarters, i.e., the two common meat products, as specific experimental products, and set three attributes connected with animal welfare (i.e., breeding time, breeding model, and diet cleanliness label) and price attributes to discuss whether each attribute has a decoy effect in the purchase behaviors of livestock and poultry products of the consumers, and compared the difference between the decoy effects of the same attribute in different products. The main conclusions are as follows: In the yellow-feathered broiler and pig hindquarter consumption experiments, setting decoy products based on the four types of attributes can all trigger positive decoy effects on the purchasing decisions of consumers. In the chicken consumption experiment, the order of effectiveness of the four types of attribute decoy was as follows: breeding model > price > diet cleanliness label > breeding time. In the pork consumption experiment, the order of effectiveness of the four types of attribute decoy was as follows: diet cleanliness label > price > breeding model > breeding time. It can be noted that in the chicken purchase decisions, the attributes that reflect cultural traditions and habits of raising broilers were more likely to trigger consumer comparison and trade-offs of decoy chicken and target chicken, while in pork purchase decisions, attributes connected to pork quality and safety were more likely to trigger consumer comparisons and trade-offs between decoy pork and target pork. Correspondingly, the breeding model of broilers and the diet cleanliness of live pigs had the highest decoy effects on consumption behaviors in relation to yellow-feathered broilers and pig hindquarters, respectively.

Accordingly, we put forward the following suggestions for government departments and related enterprises: (1) In promoting animal welfare-friendly livestock and poultry products, government departments should strengthen the promotion of the concept and levels of livestock and poultry welfare and the relationship of animal welfare to promote meat quality and safety, so that consumers can better understand the connotation and function of animal welfare attributes, such as breeding time, breeding model, and clean diet, and guide consumers not only to judge based on the price attributes when purchasing livestock and poultry meat but also to make choices based on the attribute level of animal welfare. (2) When promoting broiler products and pork products with animal welfare attributes, sellers can combine the finesse of decoy strategies to design a reasonable marketing plan for the livestock and poultry meat market. For example, appropriately setting up the chicken products with inferior breeding model attributes or inferior price attributes, and pork products with inferior diet cleanliness label attributes or price attributes, and utilizing the sensitivity of consumers to target attributes and the deviation of information processing mechanisms to guide the purchase choices of consumers for high-welfare meat products and encourage producers to improve the welfare of livestock and poultry.

The study also has certain limitations. First, the expansion set in this experiment contained four types of products, compared with the expansion sets containing three types of products in the studies of Sellers-Rubio and Nicolau-Gonzalbez (2015), Liu and Chen (2019), and Rogers et al. (2020), which may limit the identification of consumers of dominant products and limit the strength of the decoy effect. In addition, the method of comparative choice experiment adopted in this study is a hypothetical experiment without real monetary delivery behavior, and the stated preference of consumers for products may deviate from the actual consumption choice. Finally, our study was based on a representative city in China. The validity and applicability of our findings can be verified against a larger variety of geographical and cultural settings.

## Data Availability Statement

The raw data supporting the conclusions of this article will be made available by the authors, without undue reservation.

## Ethics Statement

The studies involving human participants were reviewed and approved by the ethics committee of Jiangnan University. The patients/participants provided their written informed consent to participate in this study.

## Author Contributions

LX proposed the research direction, designed the structure of the study, and wrote the manuscript. MY designed the questionnaire and analyzed the data. XC revised the manuscript. All authors contributed to the article and approved the submitted version.

## Conflict of Interest

The authors declare that the research was conducted in the absence of any commercial or financial relationships that could be construed as a potential conflict of interest.

## Publisher's Note

All claims expressed in this article are solely those of the authors and do not necessarily represent those of their affiliated organizations, or those of the publisher, the editors and the reviewers. Any product that may be evaluated in this article, or claim that may be made by its manufacturer, is not guaranteed or endorsed by the publisher.

## Footnotes

^1^The realistic and available products set in the decoy experiment.

^2^The products whose attributes are superior to those of the target product but are temporarily unavailable, so, in theory, consumers will shift their desire to target products.

^3^This involved the adoption of closed small cages to raise chickens; the size of the cages was about 33 cm (length) × 30 cm (width), and each cage could fit about three chickens. The chickens could not move freely.

^4^This involved the adoption of a free-range, mixed indoor and outdoor breeding mode. About 150 chickens could be raised per 100 square meters. The chickens could go in and out and eat freely.

^5^Live pigs were raised separately; the size of the limit fence was about 2.2 meters (length) × 70 cm (width), which was only slightly larger than the size of the pigs. The pigs could not move freely.

^6^This was adopted to raise ecological white pigs; about five to eight pigs could be raised per 100 square meters. The pigs could eat freely.

^7^For products that we hoped consumers would buy, the attributes of this product were used as reference points in the study to set decoy products.

^8^The remaining products in the core set except for the target product.
